# A Diagnostic Laboratory-Based Study on Frequency and Distribution of Viral Hepatitis B and C Among Sudanese

**DOI:** 10.2174/1874357901711010098

**Published:** 2017-10-31

**Authors:** Marwan M. Badawi, Alshaimaa A. Mohammed, Mohammed S. Mohammed, Mohammed M. Saeed, Elmoez Y. Ali, Ashraf Khalil

**Affiliations:** 1 Department of Microbilogy, Elrazi University, Khartoum, Sudan; 2 Almokhtabar Moamena Kamel Medical Laboratories, Khartoum, Sudan

**Keywords:** HBV, HCV, OBI, HCV genotypes, Epidemiological studies

## Abstract

**Background:**

Hepatitis B infection is an alarming public health problem. Almost two billion people of the population alive today, would have been infected at some time in their lives by hepatitis B. Hepatitis C virus is another life threatening condition, and about 425,000 deaths occur each year due to its complications.

The current study was carried out to provide care givers and health planners basic epidemiological data regarding the frequency and distribution of HBV and HCV based on age and sex during a time period of more than 5 years.

**Result:**

A total of 2109 different patients were found to be infected by HBV during the study period; 1641 (77.81%) were males and 468 (22.19%) were females with the age group of 20-39 years predominating (64%). In addition,16% of patients tested for HBeAg were found reactive.

**Conclusion:**

There were significant correlations observed between the levels of HBV DNA and ALT, AST and AFP. Regarding HCV, 70 males (54.9%) and 63 females (45.1%) were found to be infected, with preponderance of the age group 41 - 60 years and the genotype 4. Designing knowledge raising campaigns is appreciated as well as repetition of similar studies among larger populations in the following few years will help track a way to improvement.

## 1 INTRODUCTION

Hepatitis B and C are alarming public health problems worldwide. More than two billions amongst the population alive today, would have been infected at some time in their lives by the hepatitis B virus (HBV) and approximately 350 million of them are carriers, out of these, 25-30% would die as a consequence of the infection [1]. These carriers are at high risk of developing serious illnesses as liver cirrhosis or liver cancer. HBV complications kill more than one million per year worldwide, which is 2.7% of the total deaths occurring annually [2]. They also constitute a reservoir of infected individuals, who transmit the infection from generation to generation. Some groups are at high risk of contracting this disease compared with others depending on several factors like behavior and occupation [3, 4]. Hepatitis C virus is another life threatening condition, and about 425,000 deaths occur each year due to its complications. The global estimates showed that there are around 140 million chronic HCV cases, of whom 27% and 25% were reported as having liver cirrhosis and hepatocellular carcinoma respectively [5, 6]. In this context, it has to be stressed that liver cirrhosis and hepatocellular carcinoma have been reported to be of high incidence rates among Sudanese [7].

HBV is a complex, 42-nm double shelled DNA virus. It is transmitted through prenatal route, sexual transmission, contact with the infected body’s fluids and by the use of improper injection techniques. Hepatitis C virus (HCV) on the other hand, is a single stranded RNA virus which is transmitted mainly through transfusion of blood or blood products as well as sexually [8].

Sub-Saharan Africa is considered as a highly endemic area for HBV. In more recent studies, the HBV seropositivity varies between different regions of Sudan, ranging from 5 to 8.2% among different geographical areas [8-12]. However, earlier studies carried out in the 80’s and 90’s have estimated higher rates of HBsAg seropositivity of 17.5% among asymptomatic blood donors [13]. The seroprevalence of the infection among health workers in Sudan has been reported to be between 8 to 11% [14]. Regarding HCV, seroprevalence was found to be ranging from 2.2 to 4.8% among general Sudanese population, and up to 23.7% among haemo-dialysis patients [15, 16].

Substantial genetic variations occur within distinct regions in the two viruses, facilitating classification of eight and seven distinguishable genotypes A through H and 1 through 7 in HBV and HCV, respectively [17].

The inclusion of vaccination of HBV in Sudan in 2005 as part of the extended program of immunization, as well as the screening of blood and blood products since 2002 have been reported to reduce the rates of HBV infection and the carrier pool [10]. However, one of the major factors attributing to increased transmission of HBV is the lack of awareness about the prevalence, modes of transmission and preventive measures. The current study was carried out to provide care givers and health planners with basic epidemiological data about the frequency and distribution of HBV and HCV based on age and sex during a time period of more than 5 years, as well as to investigate relationships and correlations between several laboratory findings of patients which might contribute to the design of embeddings necessary for effective interpositions and preventive precautions.

## 2 MATERIALS AND METHODS

The present retrospective study was conducted at Almokhtabar Moamena Kamel Medical Laboratories in Sudan, Khartoum state. Almokhtabar laboratories are distributed in several regions in Middle East. Khartoum state branches receive around five hundred different specimens for different medical investigation inquiries daily. The data were retrieved from medical records from all Khartoum branches after the approval of laboratory administration. The current study aims at determining the frequency of viral hepatitis based on age and sex during a time period from October 2011 to January 2017. All Real Time PCR quantitative (TaqMan) results of HBV/HCV showing more than 20 IU/ml were considered as positive cases and included in the study based on the diagnosis algorithm. The data related to viral hepatitis as the viral load, ALT, AST, AFP, HBeAg reactivity and genotyping for HCV were collected and analyzed. ALT, AST and AFP levels were measured by ELISA. Serum HBsAg and HBeAg were quantified by electrochemiluminescence assay (Roche Diagnostic) and expressed in IU/ml. For HCV genotyping; (cobas^®^ HCV GT kits) was used (Roche Diagnostic). Real time PCR Primers in the kits target three different regions in the HCV genome (5’-UTR, Core, NS5B) to achieve excellent genotyping and subtyping accuracy. Occult Hepatitis B Viral Infection defined as the absence of DNA copies as well as antibodies against HBsAg was also investigated. Only the last results for following up patients were considered, as well as each positive result represents only one individual to ensure that the result is demonstrating the current status of the study population. All personal information of the study population were kept classified.

*Z* test was used to find out the differences in male and female proportions, while correlations were investigated between viral load and ALT, AST and AFP levels among all age groups in both sexes. *Chi* square and *T* tests were used to investigate the significance of the differences of frequencies and to determine the relationships between HBeAg reactivity and ALT, AST and AFP serum levels, respectively. Infection prevalence was not investigated as to avoid the bias in data as only the suspected patients will be preceded for HBV DNA and/or HCV RNA quantitative tests. All statistics were conducted using SPSS version 21.0 [18].

## 3 RESULT

**Fig. (1):**
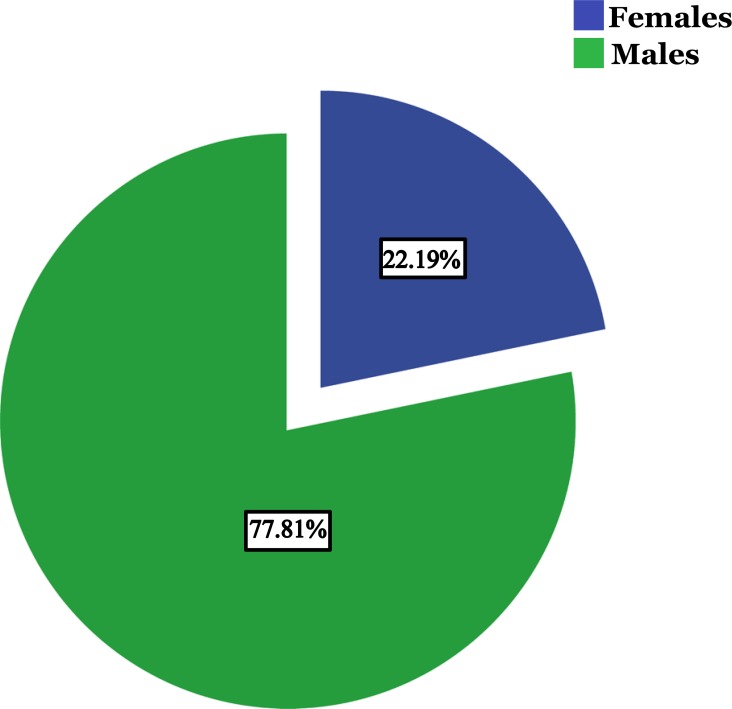
Percentage of HBV infected males and females in the study.

**Fig. (2):**
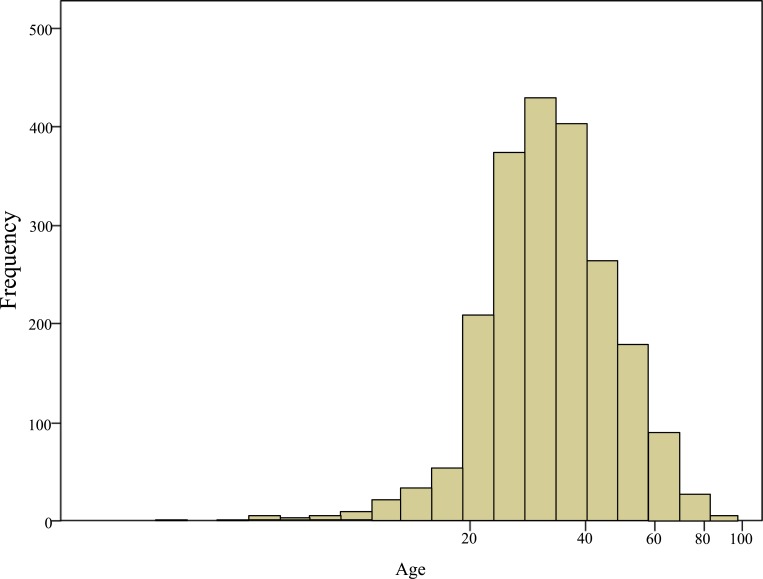
Age wise distribution of HBV infected patients.

A total of 2109 different patients were found to be infected with HBV during the study period. 1641 (77.81%) were males and 468 (22.19%) were females. Out of 2109 reported hepatitis B cases, 130 cases (6%) were in the age group of < 20 years, 1351 (64%) were between 20-39 years old, 518 (24.8%) cases were between 40-59 years old, 104 cases (5%) were between 60-79 years old and 6 cases (0.2%) were found belonging to the age group of 80 years and above. The highest proportion of cases was observed in the age group of 20-39 years, while the lowest proportion was seen among 80 years and above (Fig. **1** & **2**).

The dominance of males was observed in all age groups. As shown in Table **1**, <20 years, 69% were males and 31% were females. In the age group of 20-39 years, 80% were males and 20% were females. In 40-59 years age group, 76% were males and 24% were females. In 60-79 years age group; 64% were males and 36% were females. In the age group of 80 years and above, 4 cases were males while 2 cases were females. The age groups < 20 years (P = 0.01), 20-39 years (P = 0.00) and 60-79 years (P = 0.00) showed significant differences between males and females.

A total of 330, 277 and 88 HBV patients were found to be tested for ALT, AST and AFP, respectively [data not shown]. There were significant (at 0.05 confidence level) correlations observed between the levels of HBV DNA and ALT, AST and AFP Levels (r = 0.10, P = 0.04), (r = 0.14, P = 0.01), (r = 0.22, p = 0.03), respectively.

Relationships between HBeAg reactivity and ALT, AST and AFP levels were investigated, HBeAg reactivity was found to significantly influence the increasing level of all three liver function markers in both sexes (t = -3.854, p = 0.00), (t = -2.883, p = 0.00) and (t = -2.510, p = 0.01) respectively. No significant differences were observed between different age groups.

For determining OBI; 690 patients were found to be showing DNA copies less than 200 IU/ml in all age groups [data not shown]. Only 14 were found to be tested for the presence of HBsAg. Twelve (86%) of the 14 tested showed HBsAg reactivity, while 2 patients (14%) showed non-reactive results; none of them have been found to be tested for HBcAb. Both cases are males and belong to the age interval 20 -39 years.

**Fig. (3):**
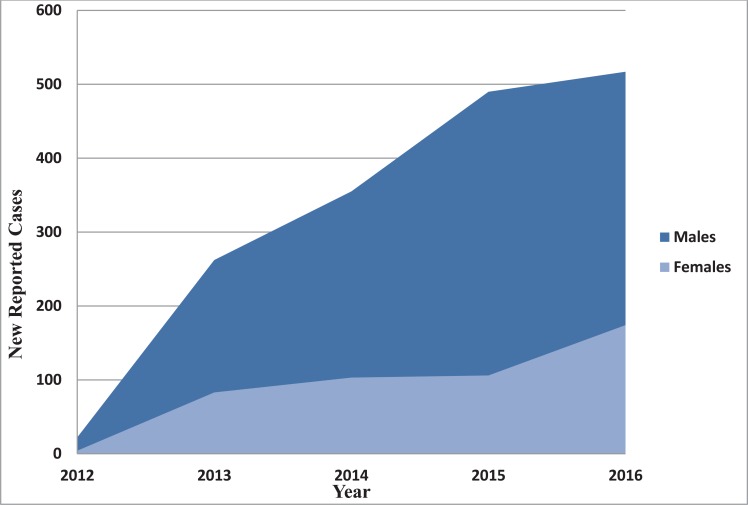
New reported cases of HBV infection in males and females.

Tremendous uptrend is observed for new reported cases of HBV of males during the study duration. New 490 and 517 males were diagnosed with hepatitis B viral infection in 2015 and 2016, respectively. Trend of new cases of both males and females from 2012 to 2016 is illustrated in (Fig. **3**).

Regarding Hepatitis C viral infection; only 133 patients were reported as infected; 70 were males (54.9%) and 63 were females (45.1%). Preponderance of the age group 41 - 60 years was observed as 68 patients out of 133 (51%) followed by 20 - 39 years as 31 patients (23%) Fig. (**4** & **5**). Genotyping of HCV was found to be investigated among 57 patients, 30 males and 27 were females Table **3**. Genotype 4 and its subtypes were found predominating Fig. (**6**). No significant differences were detected between both sexes. Only 408 participants were found to be tested for the quantification of both HBV/HCV genetic levels, among which, two cases (0.4%) were found to be infected by HBV and HCV.

For the selection bias observed, it was found to be of poor significance to provide the prevalence of the infection based on the total number of HBV/HCV laboratory inquiries.

## 4 DISCUSSION

Viral hepatitis is reported from almost all the globe and considered as an international public health problem [19]. HBV has caused frequent epidemics in Asia and Africa and endemics in Eastern Europe, the Mediterranean, South America, China and India [20-22].

Out of 2109 cases of confirmed HBV infection, 77.81% were males and only 22.19% were females. This predominance of males is reported from all over the world in similar studies including Sudan [23-32].

The highest frequency of cases of HBV occurred 64% in 20-39 years followed by 40-59 years and <19 years as 24% and 6% respectively. In the age group of 60 -79 years the proportions of cases were 5% and less infection were reported in 80 years and above which was 0.2% only. No study - to our knowledge was conducted among Sudanese determining the age wise distribution of HBV infection. However, results from different regions are found to support this finding [29, 33-35].

Furthermore, out of 98 HBeAg positives, 60 (61% from the total HBeAg positives) are in the age group 20-39, 50 and 10 males and females respectively as illustrated in Table **2**, indicating the necessity of designing effective interventions and preventive measures among this age group particularly. Sixteen percent of both males and females tested for HBeAg in all HBV infected patients are found to be positive, which highlights the acute phase as well as the high infectivity of the infection.

The levels of the three liver function markers tested are found to be significantly correlated to the viral load, as well as to HBeAg reactivity. These findings contradict the finding of Shao *et al* [36] in their study of relationship between hepatitis B virus, DNA levels and liver histology in patients with chronic hepatitis B, hence they report correlation between viral load and ALT but not AST among HBeAg negatives only.

Liver biopsies are better compared to serum regarding detection of OBI or S-escape mutants' infection. However, indication probabilities from serum samples are not to be underestimated [37, 38]. (14%) among patients of HBV DNA levels below than 200 IU/ml and tested for HBsAg in this study are found to be non reactive. Different reports suggest that OBI could be responsible for the acceleration of chronic complications and interference with treatment response [39-41].

The up trending of HBV new cases shown in Fig. (**3**) contradicts with the study conducted by Abdo *et al* [42]; Prevalence of hepatitis B virus among blood donors and assessment of blood donor’s knowledge about HBV in Sudan, as they conclude the rates of infections in 2015 to be lower than rates from 2014 - 2012. However, the difference between Abdo and colleagues study and ours is that the study population of the current study is considering all age groups as well as both sexes.

For HCV, which most of its cases are in Africa [43, 44]; the high prevalence was for males as well. However, females are as high as 45.1% of the infected cases compared to 54.91% in males as illustrated in Fig. (**4**). Osman *et al*. [33] in their study prevalence of hepatitis B surface antigen and Hepatitis C virus antibodies among pre-surgery screened patients in Khartoum, Central Sudan conclude the same finding as males predominate (60% of HCV cases). Nevertheless, several studies in different regions demonstrate the predominance of HCV infection in females rather than males [35, 45, 46]. In the neighboring Ethiopia Abel Girma Ayele and Solomon Gebre-Selassie in their study prevalence and risk factors of hepatitis B and hepatitis C virus infections among patients with chronic liver diseases in public hospitals in Addis Ababa, Ethiopia demonstrate the female predominance in HCV infected cases [28].

Predominance of the age group 41 - 60 years does accompanies - to some extend with the result of Amjad Ali *et al*. [44] in their study titled: Prevalence of active HCV virus infection in Mansehra district of Pakistan as they demonstrate the age interval 51 - 60 to be predominating. The age group of 20-39 years which we found representing only (23%) has been reported to be the most common in other studies [35, 46].

Much effort has been undertaken to establish therapeutic strategies against HCV infection. Both duration of and continuous response to the current standard therapy regimens are strongly associated with the HCV genotype [47-50]. Fifty Seven HCV cases in the study distributed in genotypes 1, 2 and 4. The majority of genotype 4 and its subtypes present in this study (89%) is known to be the most prevalent genotype in North Africa, the Middle East, and central and eastern Sub-Saharan Africa, and its prevalence has been increasing in Europe [51-53].

Subtypes of genotype 4 varies from region to region, subtypes (4c - 4e and 4h) have been reported in this study Table (3), while 4c and 4d are the predominating. The same finding has been reported in Saudi Arabia [53]. Accompanying and refuting these finding, Egypt accounts for approximately 90% of HCV infections by the genotype 4, with subtype 4a predominating [52].

## CONCLUSION

The lack of knowledge is observed in all groups irrespective of their education levels in Sudan regarding HBV and HCV routes of transmission as well as their complications and consequences. Organizing national knowledge raising campaigns is appreciated. As well as the repetition of similar studies among larger populations in the following few years will help track a way to improvement.

## Figures and Tables

**Fig. (4):**
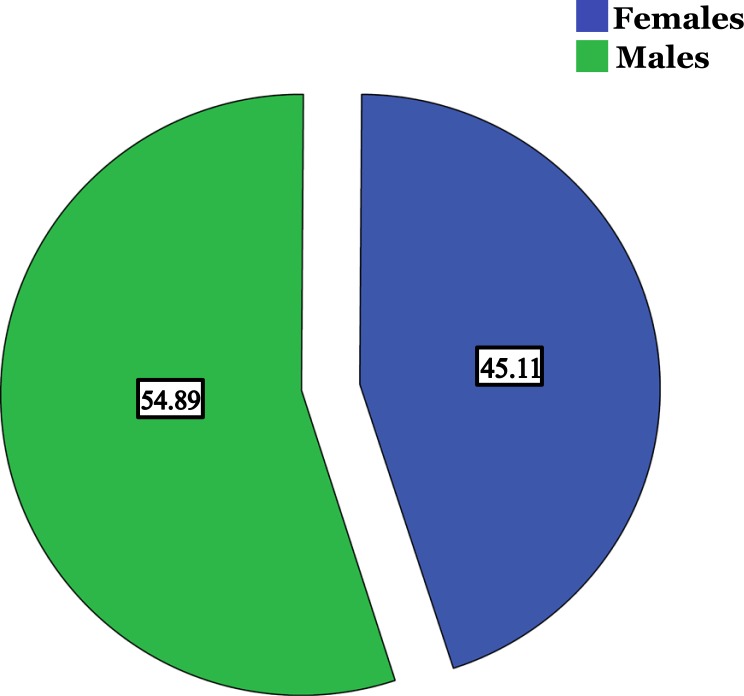
Percentage of HCV infected Males and Females in the study.

**Fig. (5):**
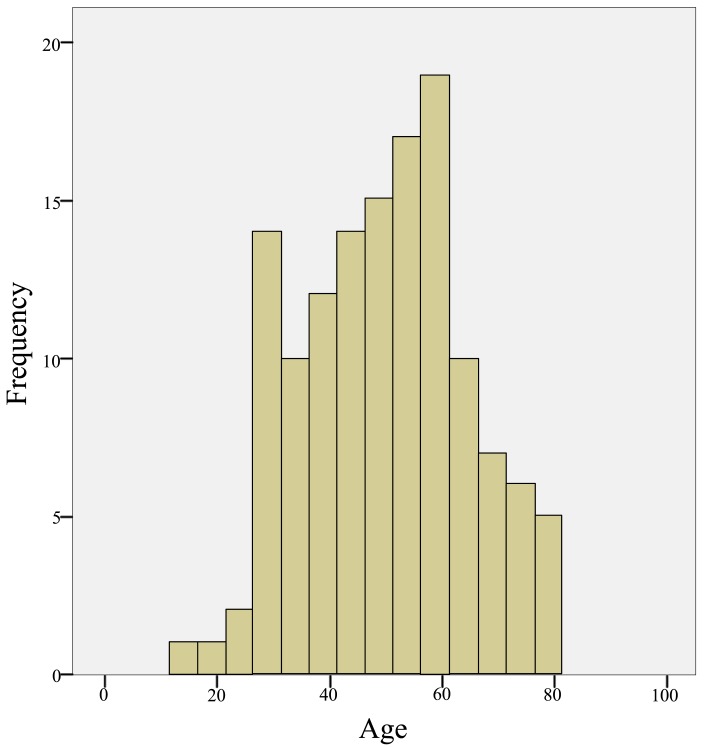
Age wise distribution of HCV infected patients.

**Fig. (6):**
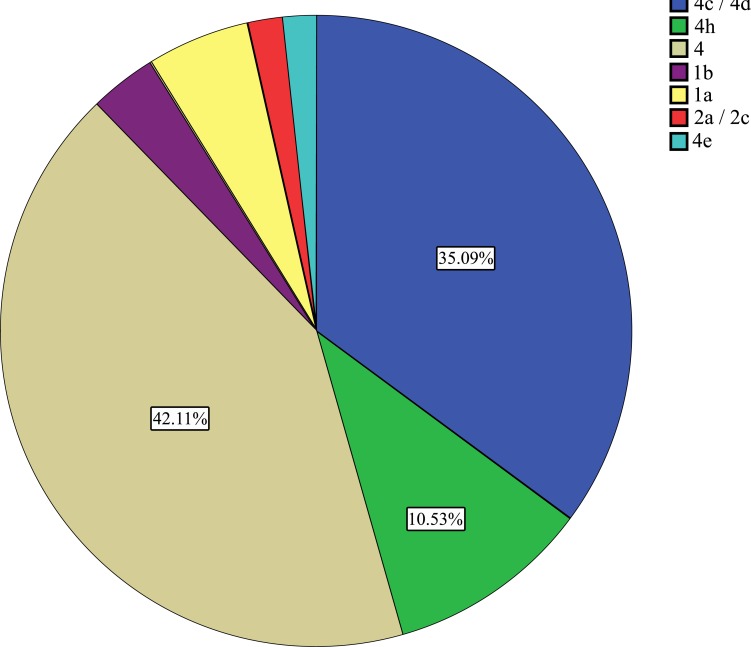
Age wise distribution of HCV infected patients.

**Table 1:** Sex and Age wise distribution of HBV infected patients.

**Age Group**	**Males** (Percentage within the Age Group)	**Females** (Percentage within the Age Group)	**Total** [Percentage within all Age Group]
**< 20 Years**	90 (69)	40 (31)	130 [6]
**20 - 39**	1086 (80)	265 (20)	1351 [64]
**40 - 59**	394 (76)	124 (24)	518 [24.8]
**60 - 79**	67 (64)	37 (36)	104 [5]
**> 80 Years**	4 (67)	2 (33)	6 [0.2]
**Total** [percentage within all age group]	1641 [78]	468 [22]	2109 [100]

632 HBV patients were tested for HBeAg from both sexes in all age groups, 74 males and 24 females were found reactive. 16% of patients tested for HBeAg were found reactive and there were no significant differences of the proportions of males and females in all age groups. All the results are summarized in (Table **2**).

**Table 2:** Sex wise distribution of HCV genotype frequencies.

Age Group	Males (Percentage within the Age Group)	Females (Percentage within the Age Group)	Total [Percentage within all Age Groups]
< 20 Years	11 (42)	9 (60)	20 [49]
20 - 39	50 (16)	10 (13)	60 [16]
40 - 59	11 (8)	4 (8)	15 [8]
60 - 79	2 (11)	1 (6)	3 [8]
> 80 Years	0	0	0
Total	74 [16]	24 [16]	98 [16]

-Data {within braces} are the percentage of the corresponding number within the HBeAg tested patients in the corresponding sex in all age groups.

**Table 3:** Sex wise distribution of HCV genotype frequencies.

Sex			Genotyping Total						
			4	4c / 4d	4h	1b	1a	2a / 2c	4e
	Male	12	10	3	0	2	0	0	27
	Female	12	10	3	2	1	1	1	30
Total		24	20	6	2	3	1	1	57
